# Exploring cluster formation in uranium oxidation using high resolution X-ray spectroscopy at elevated temperatures

**DOI:** 10.1038/s43246-025-00795-2

**Published:** 2025-04-17

**Authors:** Elena F. Bazarkina, Stephen Bauters, Yves Watier, Stephan Weiss, Sergei M. Butorin, Kristina O. Kvashnina

**Affiliations:** 1 Institute of Resource Ecology, Helmholtz-Zentrum Dresden-Rossendorf (HZDR), Dresden, Germany; 2 The Rossendorf Beamline at ESRF, The European Synchrotron, CS40220, Grenoble, France; 3 ESRF, The European Synchrotron, CS40220, Grenoble, France; 4 Condensed Matter Physics of Energy Materials, X-ray Photon Science, Department of Physics and Astronomy, Uppsala University, Uppsala, Sweden

**Keywords:** Electronic properties and materials, Electronic structure of atoms and molecules

## Abstract

Uranium dioxide (UO_2_) is a complex material with significant relevance to nuclear energy, materials science, and fundamental research. Understanding its high-temperature behavior is crucial for developing new uranium-based materials and improving nuclear fuel efficiency in nuclear reactors. Here we study the evolution of uranium state during the oxidation of UO_2_ in air at temperatures up to 550 °C using the in situ X-ray absorption spectroscopy in high energy resolution fluorescence detection mode at the U M_4_ edge, combined with electronic structure calculations. Our data reveal a complex sequence of events occurring over minutes and hours at elevated temperatures, including changes in the electronic and local structure, *5f* electron occupancy, the formation of U cuboctahedral clusters, and the creation of U_4_O_9_ and U_3_O_7_ mixed U oxide phases. These findings highlight the fundamental role of clustering processes and pentavalent uranium in both the oxidation process and the stabilization of uranium materials.

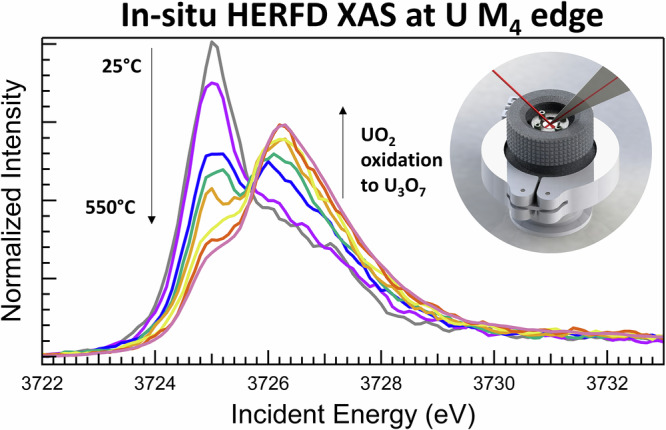

## Abstract (web summary)

.Insight into the high-temperature behavior of uranium dioxide is essential for both technological applications and fundamental studies. This work examines the evolution of uranium’s oxidation state during the thermal oxidation of uranium dioxide using U M_4_ edge X-ray absorption spectroscopy in high energy resolution fluorescence detection mode.

## Introduction

Since their discovery, actinide-containing materials^1^ have fascinated scientists due to their diverse chemical and physical properties. This diversity, coupled with actinides’ ability to exhibit multiple oxidation states and different structures, stems from the rich electronic structure of actinide atoms. Among them, uranium^2,3^ is particularly significant, forming the basis of our understanding of actinides due to its abundance on Earth^4^ and its role in nuclear fuel applications^5^. By controlling the operating temperatures, a nuclear reactor that uses UO_2_ as a fuel material can efficiently produce energy over extended periods. Besides UO_2_, several other forms of uranium, plutonium, and mixed oxide fuel materials can be used in nuclear reactors.

Significant scientific effort has been invested in exploring various actinide fuel materials under oxidation^6–8^, yet many aspects of even the UO_2_ oxidation process remain enigmatic due to the complexity of the U-O system^9–21^. Identifying stable phases is challenging^22,23^, with numerous non-stoichiometric phases potentially present, coupled with phenomena where the oxidation state of U oxides is commonly mixed^13,24–30^. These factors likely explain why the oxidation process is highly sensitive to experimental conditions such as the starting oxidation state due to sintering or aging^31^, macro- vs nano-crystallinity^32^, air moisture^31^, the rate of heating^33^, and other parameters. Despite these difficulties, the importance of the U-O system pushes the limits of our knowledge, and the concept of UO_2_ oxidation is constantly advancing mainly due to the developments of novel analytical and theoretical approaches and the significant scientific interest in this system.

Uranium dioxide is a U(IV) “parent” of the mixed oxidation state U-O “family”. The fluorite structure of stoichiometric UO_2_ consists of U(IV) occupying the regular cubic sites with eight oxygen atoms in the corners of each cube^34^. This structure is flexible and capable of generating numerous derivative structures thanks to regular “holes” or U-free cubes, which can easily accommodate additional oxygen atoms (or other anions or cations). These additions promote further structural rearrangements with both short- and long-range orders^35^. As the number of excess oxygen atoms increases, pure U(IV) in UO_2_ becomes hyperstoichiometric UO_2.0+x_ exhibiting a mixed U oxidation state and formation of other mixed oxide phases, known as U_2_O_5_, U_4_O_9_, U_3_O_7_, U_3_O_8_^23,25,36,37^. The process goes through the variation of three oxidation states U(IV), U(V), and U(VI). The end-member UO_3_ is a pure U(VI) oxide. Within the range of phase transformation from UO_2_ – U_3_O_7_, the structure remains fluorite-type; beyond this, from U_3_O_8_ to UO_3_ – an orthorhombic or “uranyl-like” phase is formed. The structural transformation and behaviour of different UO_2+x_ compounds have been recently reviewed and discussed by several authors^13,15,25–30,38^.

The concept of defects and oxygen cluster rearrangements provides the most common vision of the oxidation mechanism behind modifications in fluorite-type structures^23^. Based on early neutron diffraction studies of the Willis group^39,40^, a defect cluster model was proposed and nowadays is intensively explored by various studies^22,41–48^. According to Willis et al., O atoms can form clusters in the fluorite structure based on the displacement of O atoms and creating vacancies. However, studying these clusters is challenging due to their instability and possible relations to the U oxidation state. Therefore, Willis’s concept is generally applied to partially oxidized UO_2+x_, where the probability of cluster formation is greater, their concentration is potentially high, and they exhibit at least partial ordering. Thus, the clusters were initially proposed theoretically for UO_2.11-2.13_ and later extended to UO_2.06-2.19_ systems^41^. The formation and reorganization of clusters are proposed to be transitional for the O atom migration while the U atoms remain immobile^41^. The theoretical predictions of the O migration require experimental validation. In this study, we offer fresh insights into the oxidation process of uranium dioxide under heating, utilizing unique spectroscopic measurements that probe in situ *5f* electron states of uranium atoms as they react with oxygen from the air, incorporating additional O atoms into uranium oxides.

High-energy resolution fluorescence detection (HERFD) X-ray absorption spectroscopy (XAS) at the uranium M_4_ edge has been applied here for in situ analysis of the UO_2_ oxidation to UO_2+x_ under high-temperature conditions. HERFD XAS at the actinide M_4,5_ edges is a bulk-sensitive method that can be used to probe the *5f* electronic states^24,49,50^ is often used to investigate the electronic structure and local environment of atoms in a sample by detecting the fluorescence emitted following core-level excitation. Since HERFD XAS typically provides higher energy resolution compared to traditional XAS, it enables detailed analysis of the fine structure, including the *5f* states of actinide elements, in the bulk material. HERFD XAS allows for the detection of subtle shifts in the oxidation state by measuring changes in the position, intensity, and shape of the M₄ edge XAS features, offering a direct fingerprint of uranium’s oxidation state in different local environments^24,49,50^.

**Fig. 1:**
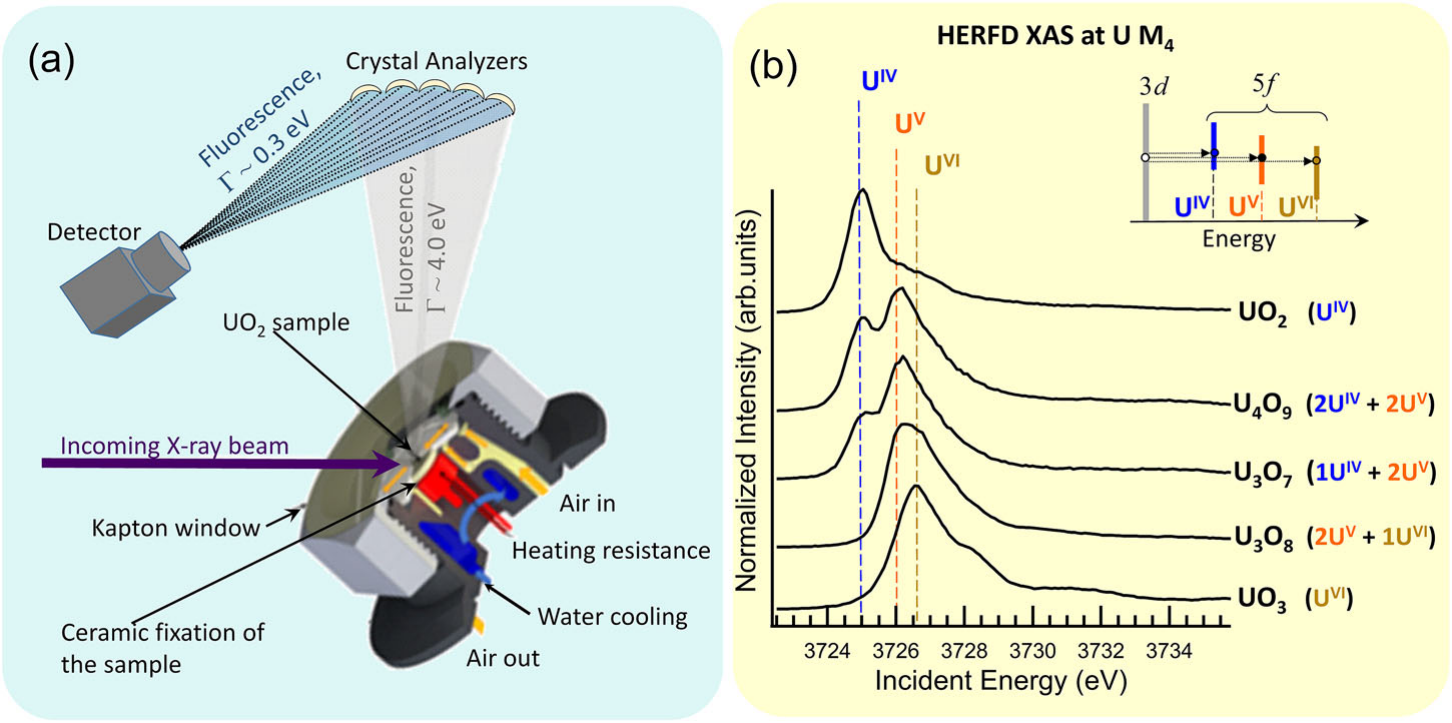
High-temperature in situ setup and basics of U M_4_ HERFD XAS methodology. **a** High-temperature cell for in situ HERFD XAS measurements at the U M_4_ edge and **b** the U M_4_ HERFD XAS spectra of uranium oxides UO_2_, U_4_O_9_, U_3_O_7_, U_3_O_8_, and UO_3_^55^. The inset in the upper right corner shows a simplified single-electron scheme of the U *3d–5f* transitions at the M_4_ edge of U for the different oxidation states.

Unlike surface-sensitive techniques such as X-ray Photoelectron Spectroscopy (XPS)^51–54^, which primarily probe the outermost layers of a sample, HERFD XAS is optimized for bulk analysis. Other methodologies, such as X-ray Diffraction (XRD) and Extended X-ray Absorption Fine Structure (EXAFS) spectroscopy at the U L₃ edge, have previously been employed in situ to investigate the formation of UO_2+x_ species^9,13,32^. While these methods are powerful for providing crystallographic information, insights into local structure, and phase transformations, HERFD XAS is uniquely suited to study electronic structures. It excels in probing the *5f* electron occupation and identifying the formation of different oxidation states in actinide systems (Fig. 1).

It is important to note that in this study, as well as in all previous studies^24,49,55,56^ utilizing the U M_4_ HERFD XAS methodology, the identification of uranium mixed states (e.g., U₄O₉, U₃O₇) has been based on the detection of uranium oxidation states. For instance, U₄O₉ comprises a 50/50 ratio of U(IV) and U(V), while U₃O₇ consists of a mixture of U(IV) and U(V) in a 33.5/66.5 ratio. This information has been determined ex situ using a fingerprint approach with the HERFD XAS technique at the U M_4_ edge^55^. We here supplement our original ex situ U M_4_ HERFD XAS study^24,55^ of uranium oxide phases with in situ measurements to track the oxidation process of UO_2_ at elevated temperatures. In situ experiments are challenging, but this HERFD XAS approach provides valuable insights into the changes in the U oxidation state throughout the process, along with corresponding alterations in the U electronic structure.

The incident and emitted X-ray energies at the U M_4_ edge (3.7 and 3.3 keV, respectively) are relatively low, making conventional thermal cells designed for hard X-ray spectroscopy unsuitable. However, the HERFD XAS method in the tender X-ray range still provides sufficient space at the sample stage to accommodate a properly constructed high-temperature reaction furnace. For this study, a custom-designed in situ cell specifically tailored for radioactive materials was developed (Fig. 1a). This pioneering in situ U M_4_ HERFD XAS experiment addresses a critical gap in our understanding of the mechanisms governing and controlling UO₂ oxidation in air, as well as the interplay between various uranium oxidation states during this process. The findings from this study provide a fresh perspective on electronic structure changes during UO₂ oxidation, with implications for nuclear energy research, environmental and materials science, and fundamental actinide chemistry.

## Results

A single experimental run has been performed by heating the UO_2_ sample up to 550 °C at the rate of 5 °C/min, keeping this *T* for 1.8 h and cooling down to ambient *T* at the same rate. The total duration of the experiment, including heating, cooling, and checking the stability of the oxidation product under ambient *T* after the experiment, was approximately 14 h. The experimental observations are described below in two parts: (1) pre-oxidation changes related to the mobility of oxygen atoms in UO_2_ at temperatures below 360 °C; and (2) the oxidation processes occurring above 360 °C. The further section is devoted to the oxidation mechanisms, the pentavalent uranium role, and U-O phase diagram.

### Pre-oxidation processes: mobility of oxygen atoms and clustering

**Fig. 2:**
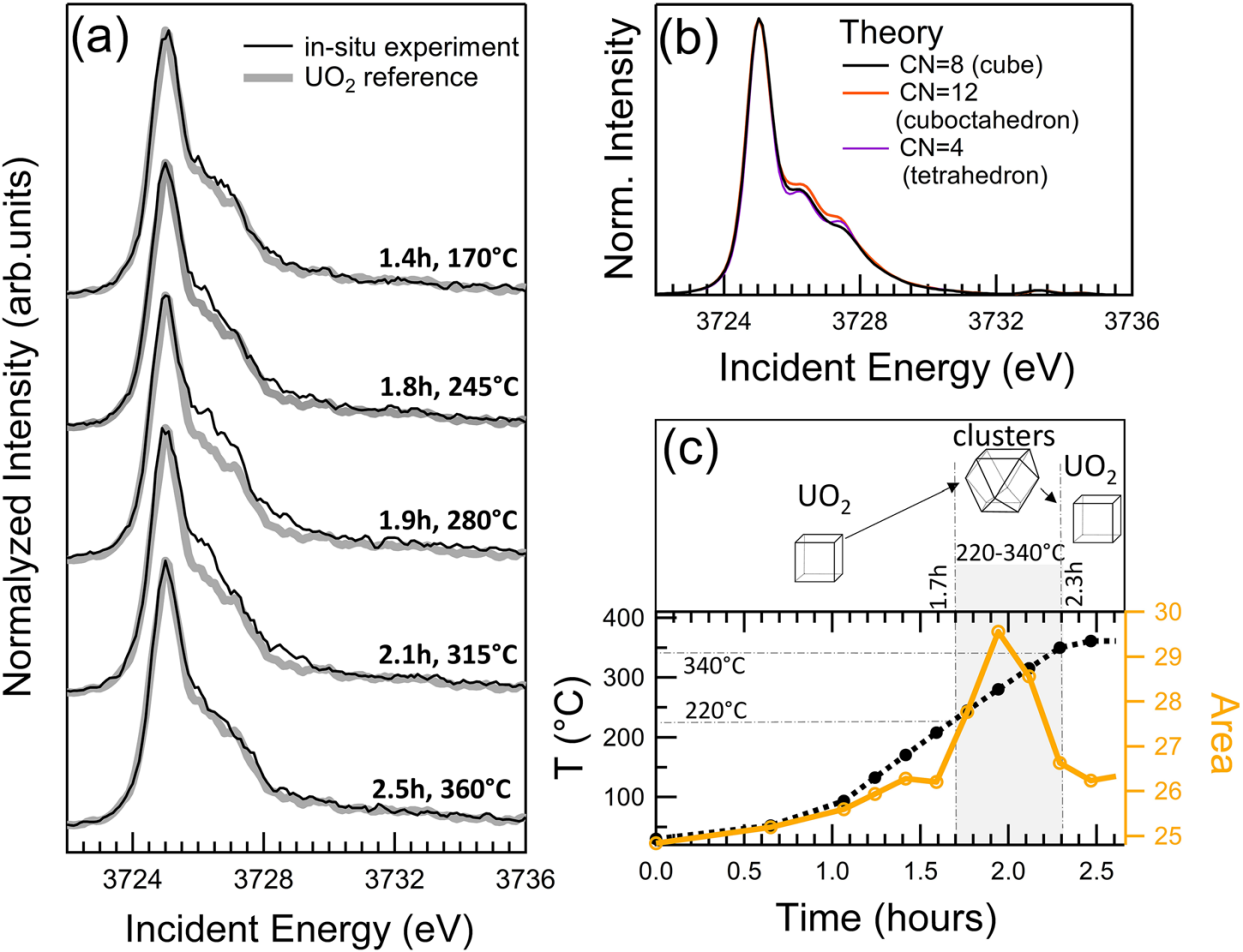
Experimental ad theoretical U M_4_ HERFD XAS data. **a** First changes in U M_4_ edge HERFD XAS spectra of UO_2_ observed with time under heating, **b** electronic structure calculations of HERFD XAS data during the clustering processes in UO_2_, **c** schematic representation of cubooctahedral clusters, temperature profile of clustering process in time, the areas of the spectra versus heating time.

During the first 1.5 h after heating begins, the U M_4_ HERFD XAS spectra show no changes up to 220 °C compared to that of the UO_2_ reference measured at room temperature, thus confirming a very high stability of UO_2_ in its ordered fluorite structure. The first changes appear after 1.8 h as the temperature rises from 220 °C to 340 °C and are summarized in Fig. 2. When normalized to the maximum, the three consecutive spectra exhibit higher intensities at the shoulder around 3725.5–3728 eV (see Fig. 2a). This corresponds to a strong progressive increase in the integrated spectral area (depicted in Fig. 2c), which is abruptly diminished at 360 °C. The next two spectra recorded at 360 °C are again identical to that of the UO_2_ reference and therefore confirm that no UO_2_ oxidation has been initiated up to 2.7 h of heating up to 360 °C. The changes observed in the spectra between 220 °C and 340 °C reflect fundamental changes in UO_2_ itself, which are discussed in detail below.

One would expect that the oxidation process goes monotonically through the mixed oxide phases. However, our experimental data reported in Fig.2a shows that an additional process takes place before the monotonic oxidation reaction starts. This pre-oxidation process is most probably related to the mobility of oxygen atoms in UO_2_ at high *T*^41,44,45^. To test this hypothesis and the sensitivity of the U M_4_ edge HERFD XAS method to this process, the electronic structure calculations of the HERFD XAS data were performed (Fig. 2b). These calculations consider changes in the uranium coordination number, ranging from 8 (in the normal fluorite structure) to 12 (in a cuboctahedron), which corresponds to the displacement of 4 oxygen atoms. In stoichiometric UO₂, if some uranium atoms become more coordinated with oxygen, an equivalent portion will have lower coordination to maintain stoichiometry. Since coordination affects the spectrum, we also examine the M_4_ edge spectrum for less coordinated uranium (CN = 4). Both experimental data and theoretical simulations show similar trends, with intensities increasing in the range of 3725.5–3728.0 eV (Fig. 2), confirming the change in U coordination while maintaining the U(IV) valence state.

The mechanism of the local O atoms reorganization (clustering) without excess O atoms involvement (i.e., without the oxidation of U(IV)) requires the local structure distortion, formation of vacancies or point defects in the fluorite structure, and initiates the clustering processes^39,41,44,57^. The temperature can influence the structure of non-oxidized UO_2_ under heating^6,58,59^. First of all, these *T* (240–350 °C) are high enough for a considerable increase of thermal vibrations inside the crystalline structure^59^. The large difference in atomic weight between O (16 amu) and U (238 amu) atoms suggests that at some point, the lighter O atoms are displaced while the heavier U atoms remain immobile. The integrated area of the spectra recorded at 245, 280, and 315 °C shows slight variations in time reflecting the variations in cluster concentration, size, or structure, while the sharp disappearance of these spectral features can be attributed to the cluster collapse. Under our experimental conditions (heating from 100 to 360 °C with the ramp of 5 °C/min), these clusters were observed for ~30 min (i.e., between 1.7 h and 2.3 h, Fig. 2c). The high stability of tetravalent U and the ordered fluorite structure makes these clusters unstable in time because no extra O atoms from the electron exchange between O_2_ and U are involved to fill up the holes in the fluorite structure. These clusters are unstable and after their collapse at 350 °C, the structure is stabilized as the normal fluorite one. Our U M_4_ HERFD XAS experimental spectrum recorded at the 360 °C is identical to the initial spectrum recorded at room temperature on UO_2_. These results supported by electronic structure calculations, show that the oxygen atom displacement and clustering are possible in non-oxidized UO_2_.

Previous experimental and theoretical studies dedicated to oxygen clusters were performed for U oxides in which U was partially oxidized, i.e., in UO_2.13_ and U_4_O_9_ in the pioneering works of Willis et al.^34,39,40^ as well as other UO_2+x_ oxides in later studies^23,37,41,42,46,48^. In those systems, the point defects are more “ordered-like” and the lifetime of the O clusters is expected to be longer. A comparison of the reference UO_2_ spectrum, the simulated one for the oxidized UO_2.13_ (as a linear combination of U(IV) and U(V) spectra), and the spectrum with clustering recorded at 280 °C is presented in Supplementary Fig. S1. The clusters observed in UO_2_ result in much weaker spectral changes in comparison with UO_2.13_. Interestingly, the possibility of structure variations in UO_2_ was recently found by in situ Pair Distribution Function (PDF) and neutron diffraction measurements under very high *T*^58^. Our results ultimately show that the local atomic displacements of O atoms are possible already at temperatures as low as 220–340 °C, but they are not stable. On the other side, these changes precede the oxidation process explained in the section below. Moreover, they are important for oxygen diffusion^60^ and thus can be the key process for oxidation. Displacements of oxygen atoms create vacancies or ‘holes’ in the fluorite structure, which facilitate oxygen diffusion. This process enhances electron exchange between O₂ molecules and uranium atoms in UO₂, leading to an increase in the O/U ratio and potentially inducing further phase transitions.

### Uranium oxidation process and phase transitions

**Fig. 3:**
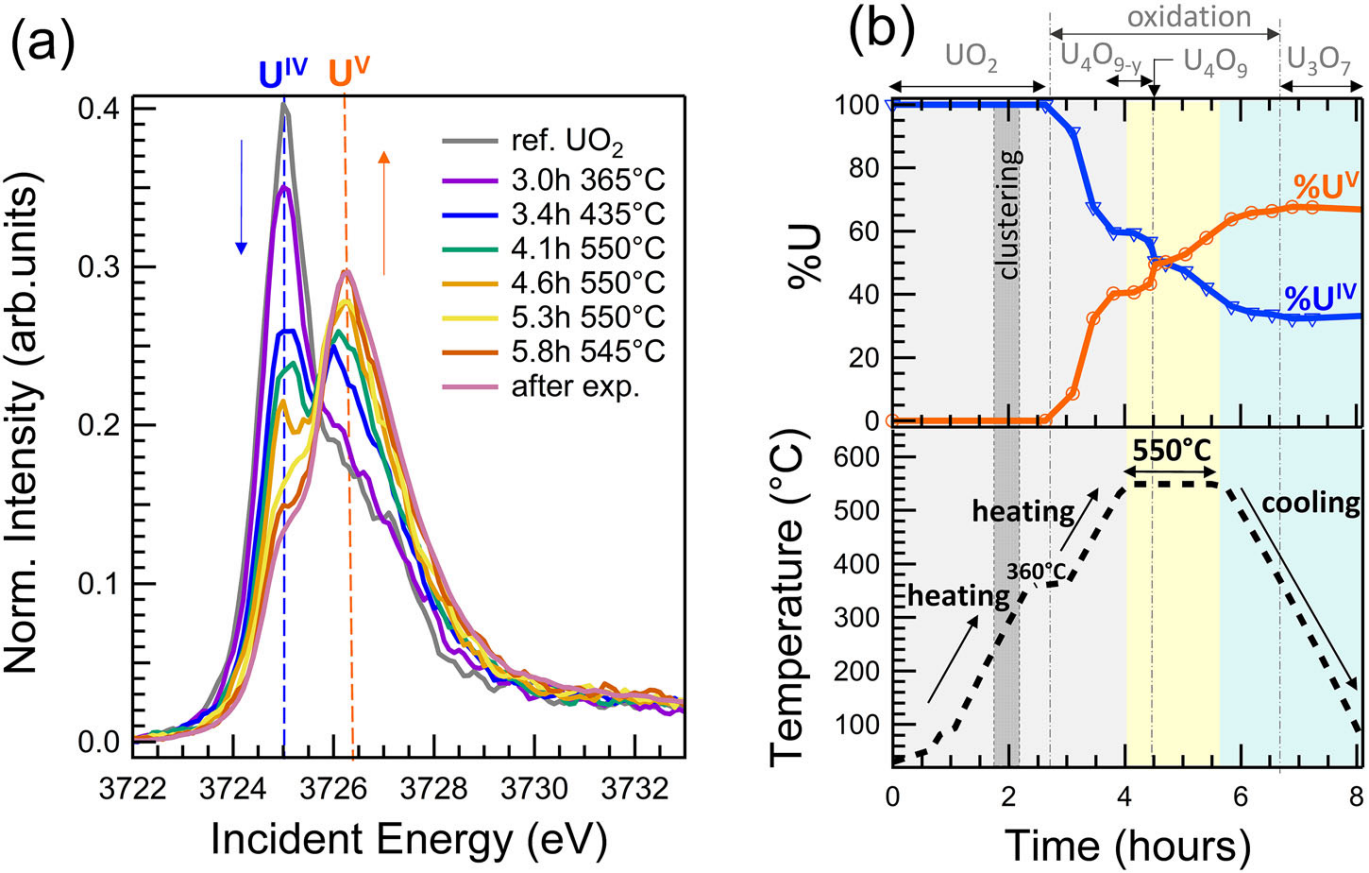
Oxidation of UO_2_ as a function of temperature and time. **a** The in situ HERFD XAS spectra at U M_4_ edge normalized to the area, **b** corresponding U(IV) and U(V) fractions, temperature profile, and the predominance of solid phases UO_2_, U_4_O_9-y_, U_4_O_9_, and U_3_O_7_. The pre-oxidation clustering process (details in Fig. 2) is indicated in (**b**) as a dotted area.

Heating from 350 °C up to 550 °C results in fast and irreversible spectra changes, indicating the dramatic changes in the U oxidation state. The main peak at 3725 eV decreases and spectra become broader with the additional peak growing at 3726.5 eV (Fig. 3a). All in situ HERFD XAS spectra were compared with the HERFD XAS spectra of well-known U_4_O_9_, U_3_O_7_, U_3_O_8_ and UO_3_ compounds (Fig. 1b). Two out of eleven spectra recorded at 550 °C are identical to that of U_4_O_9_, while all the spectra recorded under cooling at temperatures below 400 °C match those of U_3_O_7_ (Supplementary Figs. S4 and S5). U_4_O_9_ and U_3_O_7_ phases contain only U(IV) and U(V). The Principal Component Analyses (PCA) applied to all experimental spectra also found mostly two components’ contribution for the whole series (Supplementary Figs. S2 and S3). The first component is pure U(IV) (i.e., the spectrum is identical to that of the reference UO_2_), and the second component was identified as pure U(V). The pentavalent uranium compound agrees with the data of Butorin et al. ^61^, Leinders et al. ^55^, Kvashnina et al. ^49^. This spectrum of U(V) compound in the U-O system found by the Iteractive Transformation Factor Analysis (ITFA) is identical to that of UMoO_5_, which is a pure U(V) compound (Supplementary Fig. S2A). The eventual concentration of U(VI) is below the detection limit, which was estimated to be ~10% and corresponds to ~3.3% of U_3_O_8_. This means that under our experimental conditions, U(VI) does not appear during the oxidation. Our analysis indicates that the electron exchange between U(IV) and O_2_ molecules results in the formation of U(V). This U(V) is chemically bonded to the O atoms. It means that the oxidation process is accompanied by the formation of new phases with a higher O/U ratio. This agrees with previous findings that show a significant change in the chemical U-O bonding between the U(V) and U(IV) subsystems, as described within the framework of the Anderson impurity model^61^.

**Fig. 4:**
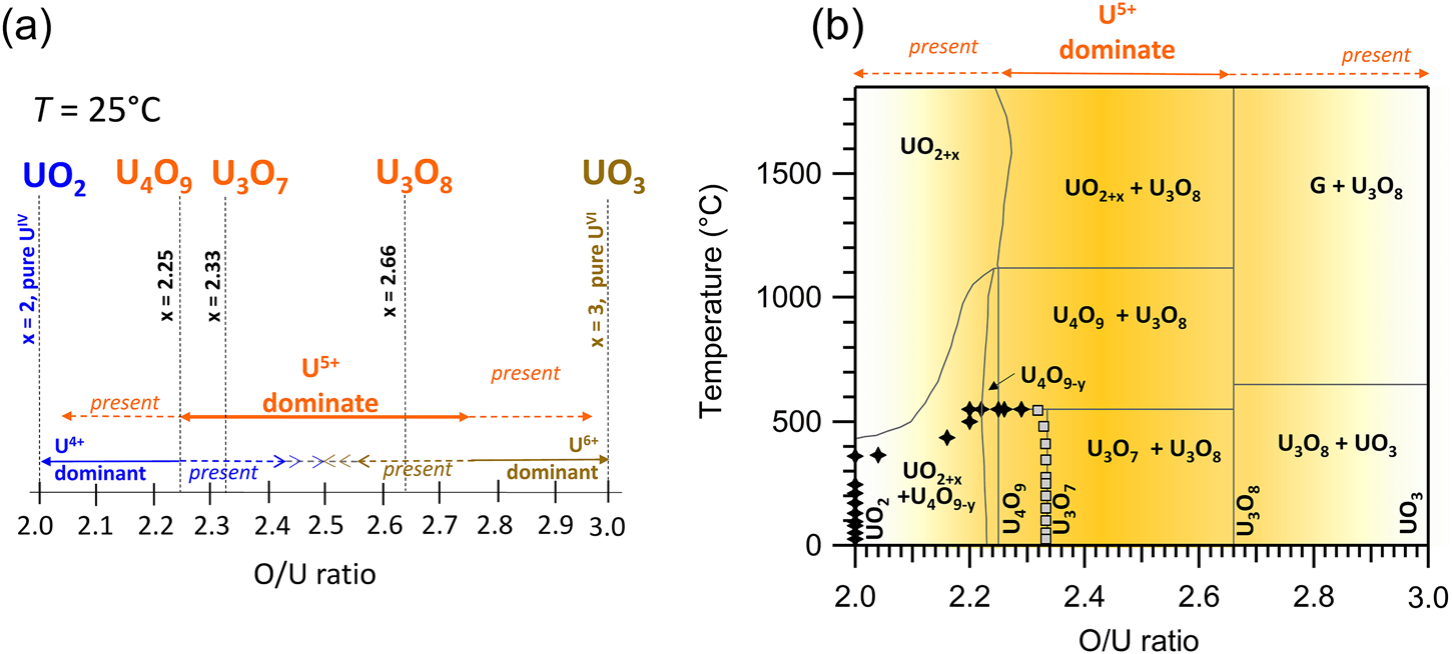
The U-O phase diagram. **a** The U(IV), U(V) and U(VI) abundance in oxides UO_2_, U_4_O_9_, U_3_O_7_, U_3_O_8_ at ambient *T* and **b** the U(V) abundance in U-O phase diagram under high *T*. U(V) is present in all UO_2+x_ phases with x above 2.0 and below 3.0 and has the biggest predominance field from *x* = 2.25 to *x* = 2.75. The phase diagram in (**b**) is a simplified version adapted from reviews^11,12,19^. The experimental data from this study are reported in (**b**) as black and blue points for the heating and the cooling, respectively.

Between 3.7 and 4.5 h of heating from 500 to 550 °C, the spectral shape stabilized (4.1 h 550 °C, Fig. 3a), with five consecutive scans showing no changes (Supplementary Fig. S4A). The oxidation state was quantified as approximately 60% U(IV) and 40% U(V). Comparing this experimental spectrum with reference compounds U_4_O_9_, the differences indicate a more reduced oxidation state (Supplementary Fig. S3A). This spectrum is assigned to non-stoichiometric U_4_O_9-y_, a likely phase in the U-O phase diagram (Fig. 4b). Interestingly, the formation of stoichiometric U_4_O_9_ (50% U(IV) and 50% U(V)) appears as a distinct kinetic process at 550 °C, with a step-like change in U(IV) and U(V) fractions (Fig. 3b). This phase predominates briefly, around 20 min between 4.6 and 4.9 h at 550 °C, as shown by two individual scans (Supplementary Fig. S4B, with the average spectrum in Fig. 3 in green as 4.6 h 550 °C).

After the brief predominance of U_4_O_9_, the oxidation process continues with the U_4_O_9_ to U_3_O_7_ phase transition (spectra after 4.6 h 550 °C, Fig. 3a). The changes occur linearly overtime at 550 °C and slow down upon cooling (Fig. 3b). Spectra recorded below 300 °C are all identical, featuring characteristics of U_3_O_7_ (Supplementary Fig. S5B). This phase contains 66.5% U(V) and 33.5% U(IV), with an O/U ratio of 2.33. The absence of U_3_O_8_ and UO_3_ spectral features confirms that U(VI) does not form during present in situ conditions. The U_3_O_7_ was monitored by U M_4_ HERFD XAS for five hours at ambient temperature, showing no changes and confirming its stability.

### U-O system: the missing puzzle pieces and the fundamental role of U(V)

Our experiments confirm that U(V) is by far the dominant oxidation state in the U-O system, which is confirmed by the U-O phase diagram reported in Fig. 4. The absence of U(VI) and U_3_O_8_ in the oxidation product of UO_2_ in our experiment is quite unexpected, given the high stability of this phase. Many studies have identified pure U_3_O_8_ as the most reliable oxidation product under high temperatures^31,32,62,63^. The U_3_O_7_ phase is less frequently reported as a final oxidation product^22,32,64^. Although U_3_O_7_ as a single stable phase was proposed in 1947^65^, it remains poorly characterized. For instance, the first U_3_O_7_ structure refinement based on neutron scattering was only published in 2021^22^. This structure is quite complex, with the U atom positions similar to those in the UO_2_ fluorite structure and the O atom positions in cuboctahedrons. This phase features an anion-excess fluorite-type structure with long-range periodic order and defects characterized by a cuboctahedral oxygen cluster arrangement. Thus, the clustering process related to increased oxygen atom mobility during heating promotes the formation and stabilization of U₃O₇. The well-crystalline U₃O₇, formed through the self-organization of cuboctahedrons, discourages the transition to U₃O₈, which is incompatible with cuboctahedrons due to its layered structure and seven-fold oxygen coordination in pentagonal bipyramids. The structural difference between U₃O₇ and U₃O₈ is significant and is often described in terms of a dramatic volume expansion (36%). However, concerning the oxidation state of uranium, both phases mainly contain U(V). Moreover, both phases have the same percentage of U(V) (66.5%), with the remaining portion (33.5%) attributed to U(IV) in U_3_O_7_ or U(VI) in U_3_O_8_. HERFD XAS data at the U M_4_ edge shows a distinct difference between U_3_O_8_ and U_3_O_7_ spectral profiles. 

## Discussion

Lewis and co-authors^13^ have recently highlighted the complexity of the U-O system, with an O:U ratio ranging from 1.5 to 3.0. Mixed oxides produced by different research groups exhibit varying crystallographic behaviours, and there is currently no consensus on the number of crystallographically distinct phases. Reports vary, citing 16 phases between UO₁.₅ and UO₃, while others suggest as many as 22 phases originating from UO_2_ alone. While the HERFD XAS methodology at the U M_4_ edge is not sensitive to crystallographic details, it provides definitive insights into uranium *5f* electron occupation and the presence of mixed oxidation states in uranium compounds. We believe that in situ HERFD XAS study at the U M_4_ edge advances our understanding of oxidation in the U-O phase diagram and sets a new benchmark for future investigations. The findings are of broad interest, not only to the materials science community but also to researchers focused on nuclear fuel corrosion, stability, and actinide chemistry^2,66^. By employing in situ HERFD XAS at the U M_4_ edge, we have enabled a unique level of detail in analyzing the oxidation processes of UO_2_. The relatively short acquisition times and exceptional sensitivity of this U M_4_ HERFD XAS method to the uranium chemical state make it a powerful tool for exploring actinide materials behavior under elevated temperatures.

Our in situ measurements reveal that UO_2_ oxidation is a complex, non-linear process characterized by distinct stages of transformation. It should be noted that previously reported ex situ results are very valuable and rich in obtained information^22,25–27,29–31,38,67–70^. They often yield results that vary due to differences in experimental parameters and the grain size of UO_2_ powders. The methodology presented here overcomes some limitations, allowing for a real-time investigation of oxidation dynamics^9,32^. The experiments were conducted under static air conditions, as detailed in the “Methods” section, and employed a controlled temperature ramping protocol. We observed the presence of clusters in non-oxidized UO₂ that exhibit oxygen atom displacements. These clusters represent a transitional state crucial for oxygen diffusion and subsequent oxidation. Their existence helps explain UO₂’s unusual thermodynamic properties, such as deviations from ideal heat capacity trends and non-linear thermal expansion. However, these U(IV) clusters are inherently unstable and collapse over time. This observation could improve strategies to stabilize or exploit these clusters in applications like catalysis or advanced nuclear material synthesis.

As oxidation progresses, the process is dominated by oxygen diffusion^31,33,43,60,64,71–73^, leading to the formation of mixed uranium phases. For instance, we observed a phase with 58% U(IV) and 42% U(V), consistent with U₄O₉₋_y_. This phase remains dominant for only about 20 min before further oxidation occurs, resulting in the formation of U₃O₇. The transient nature of U₄O₉₋_y_ and its role as an intermediary phase highlights the intricate kinetics of uranium oxidation^13,63,73–76^, which are critical for nuclear waste management and the optimization of nuclear fuel cycles. Furthermore, our heating protocol aligns well with established methods for synthesizing U₃O₇^22,33,64,70,77–79^, confirming the robustness of our approach.

Importantly, our HERFD XAS experiments at the U M₄ edge show no detectable presence of U(VI) units during the oxidation process or in the final oxidation product, with a sensitivity of 5%. Interestingly, the oxidation transition from U₄O₉ to U₃O₇ during cooling was observed to proceed linearly. This linearity, combined with the transient formation of U₄O₉, suggests a potential sintering effect that could prevent the formation of the thermodynamically favored U₃O₈ phase. This insight is crucial for developing technologies aimed at controlling oxidation processes in nuclear fuels, ensuring their stability, and exploring corrosion mechanisms.

The absence of the U(VI) signal is intriguing, as many other studies have reported that U(VI) typically forms during the oxidation of UO₂^9,31–33,62,63,78^. It should be noted that under certain conditions, U(VI) may not form readily, or its formation may be suppressed. Below, we discuss several such conditions. First, if the partial pressure of oxygen is low, UO₂ may not fully oxidize to U(VI). In such cases, uranium remains in the lower U(IV) and U(V) oxidation states. Additionally, at lower temperatures, the oxidation rate of UO₂ decreases, which can hinder the formation of U(VI). Higher temperatures are typically required to overcome the activation energy barrier for oxidation to higher oxidation states. Our experiments were conducted at temperatures up to 550 °C. Furthermore, if UO₂ is rapidly cooled during the oxidation process, there may not be sufficient time for U(VI) to fully form. Instead, a mixture of U(IV) and U(V) species could be present. In addition, when UO₂ is non-stoichiometric (i.e., UO₂₋ₓ, where x is small but non-zero), oxygen vacancies in the crystal lattice may stabilize lower oxidation states (U(IV) or U(V)), preventing complete oxidation to U(VI). Finally, the particle size of UO₂ can significantly influence the oxidation process and the final oxidation states produced.

Therefore, it is worth noting that alternative heating protocols, such as variations in ramp rates, holding times, and cooling procedures, could yield different oxidation pathways and products. Additionally, the starting material - whether stoichiometric or non-stoichiometric UO₂, nanoparticles, microparticles, thin films, or single crystals - can significantly influence the outcomes of such in situ reactions^9,15,32,38,70,78,80–85^. These factors represent important avenues for future research. Moreover, probing the oxygen ligand at the K edge^28,29,49,86^ in situ under various temperatures would be a significant advancement, which can be achieved with the help of non-resonant inelastic X-ray scattering (NIXS)^87,88^. The NIXS allows for probing the ligand K edge transitions in the soft X-ray range by using incident hard X-rays^28,29,86^. This method is irreplaceable for in- situ reactions and for samples incompatible with vacuum conditions, such as actinide materials.

Overall, our study demonstrates that pentavalent uranium is prevalent and dominates across a wide range of oxygen-to-uranium (O/U) ratios, specifically from *x* = 2.25–2.75. Moreover, the cluster formation has been observed at the beginning of the oxidation process. This in situ obtained knowledge is valuable for numerous applications, including the fabrication of novel uranium materials, advancing uranium chemistry for innovative technologies, and enhancing nuclear fuel efficiency in next-generation nuclear reactors. The findings open innovative pathways for understanding and manipulating oxidation processes, with implications for both fundamental science and practical applications.

## Online methods

### Synthesis of initial UO_2_

The synthesis of initial UO_2_ powder was done according to the procedure reported previously^56^, i.e., the microcrystalline UO_2_ powder was obtained from UF_6_ by the gas-flame method, followed by annealing under reducing conditions at 600–650 °C and further sintering at 1700 °C under a H_2_/Ar stream. The reference was characterized by X-ray diffraction (XRD); the oxygen coefficient of UO_2+x_ was found to be *x* = 0.001 (similar to refs. ^56,89,90^). The low non-stoichiometry was also confirmed by HERFD XAS at U M_4_ by comparing the spectra of our initial UO_2_ with the spectra of other well-characterized UO_2_ phases^24,49,55,61^.

For the high-temperature in situ experiment, the UO_2_ powder was pressed into a pellet, placed inside the metallic holder with Kapton confinement, and transported to ROBL under anoxic conditions.

### X-ray absorption spectroscopy in high energy resolution fluorescence detection (HERFD) mode

The measurements were performed at the BM20 ROBL beamline^91^ at the European Synchrotron Radiation Facility (ESRF, Grenoble, France). The storage ring was operated in the multi-bunch filling mode at 6 GeV with a 200 mA current. The incident energy was selected using a Si(111) double-crystal monochromator. Two Si mirrors before and after the monochromator were used to collimate the beam and reject higher harmonics. Beam size was estimated to be ~30 μm (vertically) by ~2 mm (horizontally). For the measurements, the solid sample was placed vertically and rotated by 45° to the incident beam. The incident energy was calibrated using a UO_2_ at ambient T-P conditions; its M_4_ edge maximum position was set at 3725 eV. The spectra were recorded with a 0.1 eV step, and the counting time was 3 s per point. Thus, each spectrum was ~10 min in duration. Prior to measurements, the stability of the UO_2_ sample toward radiation damage induced by X-rays has been checked by a timescan of 0.1 s for 5 min. No changes in spectral profile were found.

The spectra were collected using a Johann-type X-ray emission spectrometer in a vertical Rowland geometry. The U M_4_ HERFD XAS spectra were obtained by recording the intensity of the U M_β_ emission (3337.0 eV) as a function of the incident energy. The spectrometer was equipped with five Si(220) crystal analyzers with a 1m bending radius oriented by 75°Bragg angle, and a silicon drift X-ray detector (©Ketek). A helium-filled bag was placed to fill the optical path sample-crystal analyzers-detector to reduce the absorption of the U M_β_ fluorescence signal by air. The spectrometer was aligned using fluorescence of UO_2_ excited by the incident X-ray beam at fixed energy above the X-ray absorption edge, i.e., in the non-resonant emission mode (at energy 3775 eV). During the heating and the cooling, the same point on the sample was measured. After the experiment, the additional spectra were collected at different points, and no changes in spectral profile were found, confirming a complete and homogeneous phase transition within the whole sample. This further confirms that the changes observed in the HERFD XAS spectra are attributable to the heating process rather than to radiation damage from the intense X-rays at the synchrotron.

### In situ furnace for tender X-ray range

The in situ HERFD XAS spectroscopy measurements under heating are not trivial because they require the heating element around the sample on one side and are appropriate for X-rays at 3.3–3.7 keV transparent windows on the other side. The specially designed furnace suitable for U M_4_ edge HERFD XAS measurements (Fig. 1) includes heating resistance isolated from the furnace body by the ceramic coating, a reacting gas circulation system, and a water-cooling system inside the furnace body. The large solid-angle Kapton window of the cell allows the detection of the fluorescence signal by 5 crystal analyses. The temperature calibration performed before the experiment with one thermocouple at the inert sample (boron nitride) and the other one placed on the resistance (i.e., used for the *T* regulation) allowed for the quantification of *T* gradients as a function of the resistance temperature; all *T* values are given after correction for these gradients. The temperature cell was designed for three different experimental setups: (1) static air conditions, (2) air or specific gas circulation (flow rate of 50 mL/min), and (3) slight under-pressure/vacuum conditions (down to 100 mbar). The study reported in this manuscript was conducted under static air conditions. The special ventilation system at beamline^91^ controls other experimental conditions: the humidity of the air in the experimental hutch (37 ± 5%), pressure (1005 ± 2 mbar), and the ambient temperature in the experimental hutch (23 ± 2 °C). The temperature ramp was 5 °C/min for both heating and cooling processes.

### Quantification analysis

Two types of U M_4_ edge normalization were used: normalization to 1 and normalization to the spectrum area. For quantifying U(IV) and U(V) fractions (Fig. 3), normalization to the area was applied, ensuring that the sum of components in the linear combination was 100% within 2% (Supplementary Table S1). Normalization to 1 (i.e., to the maximum) was used for analyzing the clustering process (Fig. 2). The quantification of U(IV) and U(V) fractions is based on Factor Analysis^92^. A data matrix is constructed with the experimental points of spectra. The spectral components are called factors.

In the first step, eigenanalysis, a mathematical technique used in linear algebra to study linear transformations, is applied to determine the rank, i.e., the number of linearly independent spectral components (Supplementary Fig. S2). This is achieved by solving an eigenvalue problem using the covariance matrix. The eigenvalues and eigenvectors obtained from eigenanalysis provide an orthogonal basis for the factor space, representing the pure components and their spectral signatures. Typically, only a few primary eigenvectors account for significant data variation, while others, caused by experimental errors, are neglected (Supplementary Fig. S2B). The number of independent factors is identified using the eigenvalues and a semi-empirical indicator function. The number of primary factors is determined when the indicator function reaches its minimum value.

In the second step, the number of pure components was fixed to two: U(IV) and U(V). All spectra were quantified using Iterative Target Testing (ITT), which involves rotating the orthogonal basis of the factor space represented by the eigenvectors. The ITT procedure uses concentration test vectors for non-orthogonal rotation and determines factor concentrations independently. Based on comparisons with reference compounds, the initial UO_2_ sample was assumed to contain 100% U(IV), the sample at 4.7 h 550 °C was assumed to contain 50% U(IV) and 50% U(V), and the oxidation product was assumed to contain 33.5% U(V) and 66.5% U(VI). Other spectra had unknown distributions of U(IV) and U(V). ITT results for the experimental spectra are provided in Supplementary Table S1, and Fig. S3 shows the spectra of the individual compounds U(IV) and U(V) in comparison with independently measured reference compounds.

### Electronic structure calculations

To obtain the HERFD XAS spectra at the U M_4_ edge, the core-to-core (3d–4f) resonant inelastic X-ray scattering intensity maps were calculated on the emission versus incident photon energy scales and a cut at the constant emission energy, corresponding to the maximum of the RIXS intensity was made along the incident photon energy axis. The RIXS maps were calculated using the crystal field multiplet theory approach as described in detail in ref. ^93^. The Slater integrals F^2,4,6^ (*5f,5f*), F^2,4^ (*3d,5f*), F^2,4,6^ (*4f,5f*) as well as G^1,3,5^ (*3d,5f*) and G^0,2,4,6^ (*4f,5f*) calculated for the U(IV) ion were scaled down to 80% of their ab-initio Hartree–Fock values in the computation of the RIXS maps. The values of Wybourne’s crystal field parameters in O_h_ symmetry (the case of the UO_2_ reference) were set to B^4^_0_ = −0.93 and B^6^_0_ = 0.35 eV. U has coordination number 8 in UO_2_. To calculate the spectra of other U(IV)-O polyhedra with U coordination numbers 12, the change in the crystal field effect was accounted by setting the crystal field parameters according to relations between cubic and cuboctahedral phases B^4^_q_[cuboctahedron] = (−1/2)B^4^[octahedron] and B^6^_q_[cuboctahedron] = (−13/4)B^6^_q_[octahedron] as described in ref. ^94^. The ground, intermediate, and final states of the spectroscopic process were represented by the 3d^10^5f^2^, 3d^9^5f^2+1^, and 4f^13^5f^2+1^ configurations, respectively.

## Supplementary information

Supplemental Information

## Data Availability

The data that support the findings of this study are available from the corresponding author upon request.
