# Supplementary material for: Exploring cluster formation in uranium oxidation using high resolution X-ray spectroscopy at elevated temperatures
Source: Commun Mater. 2025 Apr 17;6(1):75. doi: 10.1038/s43246-025-00795-2 (PMC12006023; doi:10.1038/s43246-025-00795-2)
Supplement: Supplementary file 1 — Supplemental Information [file 43246_2025_795_MOESM1_ESM.docx]

SUPPLEMENTARY INFORMATION

**Exploring Cluster Formation in Uranium Oxidation using High Resolution X-ray Spectroscopy at Elevated Temperatures**

Elena F. Bazarkina^1,2^, Stephen Bauters^1,2^, Yves Watier^3^, Stephan Weiss^1^, Sergei M. Butorin^4^ and Kristina O. Kvashnina*^1,2^

*^1^Institute of Resource Ecology, Helmholtz Zentrum Dresden-Rossendorf (HZDR), PO Box 510119, 01314 Dresden, Germany*

*^2^The Rossendorf Beamline at ESRF, The European Synchrotron, CS40220, 38043 Grenoble Cedex 9, France*

*^3^ESRF, The European Synchrotron, CS40220, 38043 Grenoble Cedex 9, France*

*^4^Condensed Matter Physics of Energy Materials, X-ray Photon Science, Department of Physics and Astronomy, Uppsala University, PO Box 516, SE-75120 Uppsala, Sweden*

**email: kristina.kvashnina@esrf.fr*

**Supplementary Figure S1.** Comparison of experimental in-situ spectra (1.9h 280°C) assigned to the clustering process in UO_2_ with the spectrum of reference UO_2_ and model spectra of UO_2.13_. The model spectra of UO_2.13_ were calculated assuming the U charge to be 4.26 which corresponds to the sum of 74% of U(IV) and 26% of U(V) (i.e. 0.74 parts of the initial UO_2_ spectrum and 0.26 parts of UMoO_5_ spectrum).

**Supplementary Figure S2.** Eigenvalues as a function of eigenvector number (A) and eigenvector functions (B) found by the Principle Component Analyses (PCA) both show only 2 components, i.e. U(IV) and U(V), in the in-situ experimental spectra at 25-550°C. The spectra showing the presence of clusters are analyzed separately (Figure 2).

**Supplementary Figure S3.** Theoretical components found by ITFA in comparison with pure U(IV) and U(V) compounds (A) and the comparison of the experimental HERFD XAS U M_4_ edge spectra with the Linear combination model (B). In (B), the number of averaged scans is given in parentheses. The ITFA components were found by the PCA analyses using experimental spectra (B). Thus, UO_2_ and UMoO_5_ standards are independent.

**Supplementary Figure S4.** Comparison of in-situ HERFD XAS spectra recorded at 500-550°C with the spectrum of reference compound U_4_O_9_. (A) The spectra with stable shape recorded between 3.7-4.4h at 500-550°C that correspond to the non-stoichiometric phase U_4_O_9-y_. (B) Two spectra recorded at 4.6-4.8h 550°C are identical to the reference U_4_O_9_.

**Supplementary Figure S5.** Comparison of HERFD XAS spectra at the U M4 edge for oxidation product reference compounds, including U_3_O_8_ and β-UO_3_ (A) and U_3_O_7_ (B). Stars indicate the characteristic spectral features of U(VI), while arrows highlight the distinctive features of U­_3_O_7_.

**Supplementary Table S1.** Iterative Target Testing (ITT) quantitative analysis of U(IV) and U(V) fractions in U-O phases during oxidation as a function of time and temperature.

| ***Exp. time*** | ***T°C*** | ***Number of Scans*** | ***U(IV), %*** | ***U(V), %*** | ***Sum*** |
| --- | --- | --- | --- | --- | --- |
| *heating* |  |  |  |  |  |
| *2.5h* | *360* | *2* | *100* | *0* | *100* |
| *3.0h* | *365* | *2* | *90.5* | *8.6* | *99.1* |
| *3.4h* | *435* | *2* | *67.7* | *32.5* | *100.2* |
| *3.7h* | *500* | *2* | *59.7* | *40.2* | *99.9* |
| *4.1h* | *550* | *2* | *59.8* | *41.0* | *100.9* |
| *4.4h* | *550* | *1* | *55.9* | *42.7* | *98.7* |
| *4.5h* | *550* | *1* | *50.8* | *49.7* | *100.4* |
| *4.6h* | *550* | *2* | *50.3* | *50.6* | *100.8* |
| *5.0h* | *550* | *2* | *47.2* | *52.4* | *99.7* |
| *5.3h* | *550* | *2* | *42.2* | *57.9* | *100.1* |
| *cooling* |  |  |  |  |  |
| *5.8h* | *545* | *2* | *36.2* | *63.6* | *99.8* |
| *6.1h* | *480* | *2* | *34.3* | *65.7* | *100.0* |
| *6.5h* | *410* | *2* | *33.7* | *66.5* | *100.3* |
| *6.8h* | *345* | *2* | *32.1* | *67.0* | *99.1* |
| *7.2h* | *275* | *2* | *32.3* | *67.2* | *99.5* |
| *7.5-13.8h* | *200-30* | *32* | *33.7* | *67.2* | *100.9* |
| *error** |  |  | *±0.5* | *±0.5* |  |

*
